# Adaptive hybrid robotic system for rehabilitation of reaching movement after a brain injury: a usability study

**DOI:** 10.1186/s12984-017-0312-4

**Published:** 2017-10-12

**Authors:** F. Resquín, J. Gonzalez-Vargas, J. Ibáñez, F. Brunetti, I. Dimbwadyo, L. Carrasco, S. Alves, C. Gonzalez-Alted, A. Gomez-Blanco, J. L. Pons

**Affiliations:** 1 Neural Rehabilitation Group, Cajal Institute of the Spanish National Research Council (CSIC), Avda. Doctor Arce, 37, 28002 Madrid, Spain; 2 Catholic University of Asunción, Asunción, Paraguay; 3 Occupational Therapy Department. Occupational Thinks Research Group. Instituto de Neurociencias y Ciencias del Movimiento (INCIMOV), Centro Superior de Estudios Universitarios La Salle. Universidad Autónoma de Madrid, Madrid, Spain; 4 Occupational Thinks Research Group, Centro Superior de Estudios Universitarios La Salle, Universidad Autónoma de Madrid, Madrid, Spain; 5 Centro de Referencia Estatal de Atención al Daño Cerebral (CEADAC), Madrid, Spain; 6 Sobell Department of Motor Neuroscience and Movement Disorders, Institute of Neurology, University College London, London, UK; 7 Tecnológico de Monterrey, Monterrey, México

**Keywords:** Hybrid robotic systems, Upper limb rehabilitation, Stroke rehabilitation, Functional electrical stimulation, Feedback error learning

## Abstract

**Background:**

Brain injury survivors often present upper-limb motor impairment affecting the execution of functional activities such as reaching. A currently active research line seeking to maximize upper-limb motor recovery after a brain injury, deals with the combined use of functional electrical stimulation (FES) and mechanical supporting devices, in what has been previously termed hybrid robotic systems. This study evaluates from the technical and clinical perspectives the usability of an integrated hybrid robotic system for the rehabilitation of upper-limb reaching movements after a brain lesion affecting the motor function.

**Methods:**

The presented system is comprised of four main components. The hybrid assistance is given by a passive exoskeleton to support the arm weight against gravity and a functional electrical stimulation device to assist the execution of the reaching task. The feedback error learning (FEL) controller was implemented to adjust the intensity of the electrical stimuli delivered on target muscles according to the performance of the users. This control strategy is based on a proportional-integral-derivative feedback controller and an artificial neural network as the feedforward controller. Two experiments were carried out in this evaluation. First, the technical viability and the performance of the implemented FEL controller was evaluated in healthy subjects (*N* = 12). Second, a small cohort of patients with a brain injury (*N* = 4) participated in two experimental session to evaluate the system performance. Also, the overall satisfaction and emotional response of the users after they used the system was assessed.

**Results:**

In the experiment with healthy subjects, a significant reduction of the tracking error was found during the execution of reaching movements. In the experiment with patients, a decreasing trend of the error trajectory was found together with an increasing trend in the task performance as the movement was repeated. Brain injury patients expressed a great acceptance in using the system as a rehabilitation tool.

**Conclusions:**

The study demonstrates the technical feasibility of using the hybrid robotic system for reaching rehabilitation. Patients’ reports on the received intervention reveal a great satisfaction and acceptance of the hybrid robotic system.

**Trial registration:**

Retrospective trial registration in ISRCTN Register with study ID ISRCTN12843006.

## Background

Upper limb hemiparesis is one of the most common consequences after a brain injury accident [1]. This motor impairment has an adverse impact on the quality of life of survivors since it hinders the execution of activities of daily living. From the rehabilitation perspective, it is widely accepted that high-intensity and repetitive task-specific practice is the most effective principle to promote motor recovery after a brain injury [1, 2]. However, traditional rehabilitation treatment offers a dose of movement repetition that is in most cases insufficient to facilitate neural reorganization [3]. In response to these current clinical shortcomings, there is a clear interest in alternative rehabilitation methods that improve the arm motor functionality of brain injury survivors.

Hybrid robotic systems for motor rehabilitation are a promising approach that combine the advantages of robotic support or assistive devices and functional electrical stimulation (FES) technologies to overcome their individual limitations and to offer more robust rehabilitation interventions [4]. Despite the potential benefits of using hybrid robotic systems for arm rehabilitation, a recent published review shows that only a few hybrid systems presented in the literature were tested with stroke patients [4]. Possible reasons could be the difficulties arising from the integration of both assistive technologies or the lack of integrated platforms that can be easily setup and used.

End-effector robotic devices combined with FES represent the most typical hybrid systems used to train reaching tasks under constrained conditions [5–7]. With these systems, patients’ forearms are typically restricted to the horizontal plane to isolate the training of the elbow extension movement. The main advantage of this approach is the simplicity of the setup, with only 1 Degree of Freedom (DoF). However, to maximize the treatment’s outcomes and achieve functional improvement it is necessary to train actions with higher range of motion (> 1 DoF) and functional connotations [8, 9]. Yet, the complexity for driving a successful movement execution in such scenarios requires the implementation of a robust and reliable FES controller.

The appropriate design and implementation of FES controllers play a key role to achieve stable and robust motion control in hybrid robotic systems. The control strategy must be able to drive all the necessary joints to realize the desired movement, and compensate any disturbances to the motion, i.e. muscle fatigue onset as well as the strong nonlinear and time-varying response of the musculoskeletal system to FES [10, 11]. Consequently, open-loop and simple feedback controllers (e.g. proportional-integral-derivative -PID-) are not robust enough to cope with these disturbances [8, 12]. Meadmore et al. presented a more suitable hybrid robotic system for functional rehabilitation scenarios [13]. They implemented a model-based iterative learning controller (ILC) that adjusts the FES intensity based on the tracking error of the previously executed movement (see [13, 14] for a detail description of the system). This iterative adjustment allows compensating for disturbances caused by FES. Although this approach addresses some of the issues regarding motion control with FES, it requires a detailed mathematical description of the musculoskeletal system to work properly. In this context, unmodeled dynamics and the linearization of the model can reduce the robustness of the controller performance. Also, the identification of the model’s parameters is complex and time consuming, which limits its applicability in clinical settings [11, 12].

The Feedback Error Learning (FEL) scheme proposed by Kawato [15] can be considered as an alternative to ILC. This scheme was developed to describe how the central nervous system acquires an internal model of the body to improve the motor control. Under this scheme, the motor control command of a feedback controller is used to train a feedforward controller to learn implicitly the inverse dynamics of the controlled system on-line (i.e. the arm). Complementary, this on-line learning procedure also allows the controller to adapt and compensate for disturbances. In contrast with the ILC, the main advantage of this strategy is that the controller does not require an explicit model of the controlled system to work correctly and that it can directly learn the non-linear characteristic of the controlled system. Therefore, using the FEL control strategy to control a hybrid robotic system can simplify the setup of the system considerably, which makes easier to deploy it in clinical settings as well as personalize its response according to each patient’s musculoskeletal characteristics and movement capabilities. The FEL has been used previously to control the wrist [16] and the lower limb [17] motion with FES in healthy subjects; but it has not been tested on brain injury patients. In a previous pilot study, we partially showed the suitability of the FEL scheme in hybrid robotic systems for reaching rehabilitation with healthy subjects [18]. However, a rigorous and robust analysis has not been presented neither this concept has not been tested with motor impaired patients.

The main objective of this study is to verify the usability of a fully integrated hybrid robotic system based on an FEL scheme for rehabilitation of reaching movement in brain injury patients. To attain such objective two-step experimentation was followed. The first part consists of demonstrating the technical viability and learning capability of the developed FEL controller to drive the execution of a coordinated shoulder-elbow joint movement. The second part consists of testing the usability of the platform with brain injury patients in a more realistic rehabilitation scenario. For this purpose, we assessed the patients’ performance and overall satisfaction and emotional response after using the system.

## Methods

In this section, we present the hybrid robotic system for the rehabilitation of reaching movement in patients with a brain injury. The system focuses on aiding users to move their paretic arm towards specific distal directions in the space. During the execution of the reaching task, the FEL controller adjusts the intensities of the electrical stimuli delivered to target muscles in order to aid the subjects in tracking accurately the target paths.

### Description of the hybrid rehabilitation platform for reaching rehabilitation

**Fig. 1:**
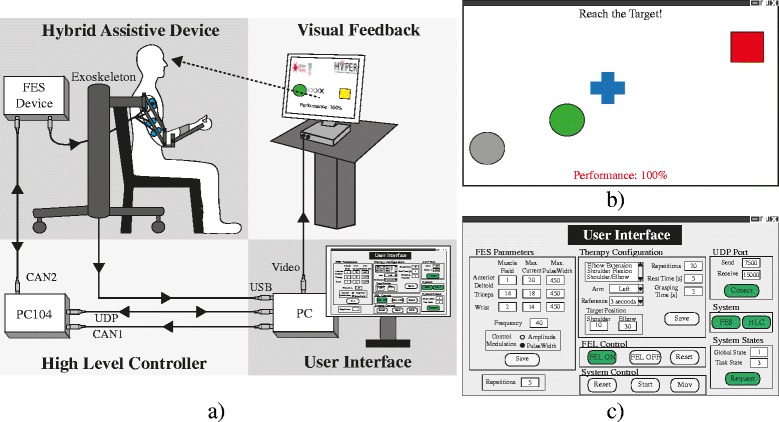
**a** General overview of the presented hybrid robotic platform for reaching rehabilitation. **b** Visual feedback provided to the users. The green ball represents the actual arm position, the blue cross is the reference trajectory, the initial and final position are represented by the gray ball and red square respectively. **c** Interface for system configuration

Figure 1 shows the general overview of the developed platform. This rehabilitation platform is composed of four main components: the hybrid assistive device (upper limb exoskeleton + FES device); the high-level controller (HLC); the visual feedback and; the user interface.

The hybrid assistance is given by the upper limb exoskeleton, Armeo Spring® (Hocoma, Switzerland) and the IntFES stimulator (Technalia, Spain). The Armeo is a passive exoskeleton aimed at supporting the arm weight against gravity. Also, the exoskeleton delimits the workspace, bounding the movements to a controlled area. Since stroke patients suffer typically from an over-activity of flexor muscles of the arm and a loss in activity of the triceps, anterior deltoids and finger extensor muscles [13, 19], the FES is delivered through biphasic electrical pulses at the triceps and the anterior deltoid muscles.

The HLC is implemented in a PC104 architecture running under xPC Target® operating system (The MathWorks Inc.) for real-time operation. This component estimates the arm joint position, generates the reference trajectory (from the initial position to the target) and executes the control algorithm to command the FES intensity delivered at target muscles.

Figure 1b shows the visual feedback interface, which is integrated into the platform to guide and encourage the user to accomplish the rehabilitation task. In order to present users an intuitive and easy to understand visualization paradigm, geometrics blocks were used to represent the arm movement on the screen and guide the rehabilitation session. Thus, the user’s arm movement is represented by a green circle, where the x- and y-axis indicate the movements of the elbow and shoulder joints, respectively. The blue cross represents the reference trajectory that users should follow. This cross moved from an initial position (grey circle) to the final position (red square).

At the end of each trial, the performance of the task is calculated and shown to the user, who is in turn instructed to maximize this result throughout the session. The performance is estimated from the difference between the generated signal reference and the current position of the controlled joints (see Eq. 4). This score is also used to change the color of the ball during the task execution. This way, the system provides an augmented feedback, which allows users to monitor their performance during the movement. The ball turns green if the performance is excellent (80% or more), yellow if it is good (between 60 and 80%), orange if it is moderate (between 40 and 60%) and red when it is poor (40% or less).

Lastly, a user interface (Fig 1c) is integrated into the architecture allowing the easy configuration of the therapy parameters, i.e. trained right/left arm, FES parameters, tracking reference velocity and range of movements. Both interfaces (visual feedback and user configuration) were coded and implemented using custom made Matlab methods.

### FES-based controller design

#### Human arm position

**Fig. 2:**
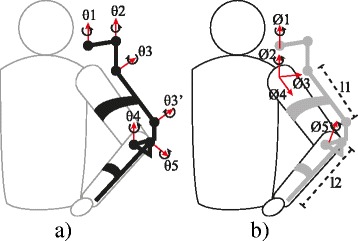
Kinematic representation of the rotation axes. **a** Exoskeleton θ = {θ_1_, θ_2_, θ_3_, θ_4_, θ_5_}. **b** Human arm Ø = {Ø_1_, Ø_2_, Ø_3_, Ø_4_, Ø_5_}

Figure 2a depicts the rotation axes of angular position transducers embedded in the exoskeleton. With these transducers, the angular position of the human arm joints can be inferred considering the following assumptions: i) there is a fixed parallel arrangement between the arm and the exoskeleton segments l1 and l2 (Fig. 2b); ii) the stimulation of the anterior deltoids produces a moment on an axis that is fixed with respect to the shoulder (axis Ø_2_), and the stimulation of the triceps produces a moment on the axis that is orthogonal to both the forearm and the upper arm (axis Ø_5_). Hence, the vector Ø = [Ø_1_, Ø_2_, Ø_3_, Ø_4_, Ø_5_], representing the human arm position, is defined by implementing the same objective transformation fully described in [20, 21].

#### Feedback error learning implementation

The main goal of the FES-based controller is to adjust the intensity of the electrical stimuli provided on specific muscles to achieve a precise control of motion. For such purpose, the FEL algorithm modulated the pulse width (PW) of the electrical pulse delivered at the anterior deltoids and triceps muscles between 50 and 450 μs. The frequency of the stimulation was 40 Hz and a constant pulse amplitude was used. The amplitude was adjusted according to the motor response and comfort of each user.

**Fig. 3:**
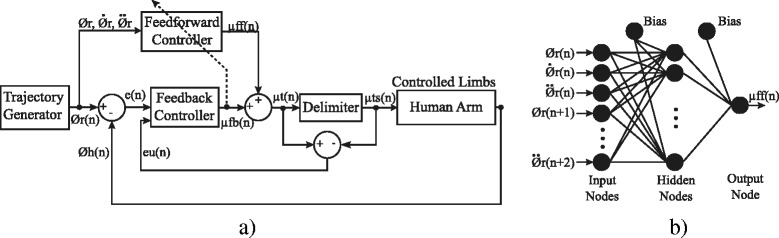
**a** Block diagram of the FES-based Feedback Error Learning (FEL) controller. **b** Artificial Neural Network used as feedforward loop. ${\mathrm{\O}}_{r,}\kern0.5em {\dot{\mathrm{\O}}}_r,\kern0.5em {\ddot{\mathrm{\O}}}_r$ represent the desired angular position, velocity and acceleration respectively; *Ø*_*h*_ is the measured position of the human arm; e(n) is the error position; *μ*_*ff*_, *μ*_*fb*_ are the control signal generated for the feedback and feedforward controllers respectively; *μ*_*t*_ is the total assistance; *μ*_*ts*_ is the assistance at the output of the saturator; *e*_*u*_ is the difference between *μ*_*ts*_ and *μ*_*t*_

In this work, two FEL controllers were implemented (one for each joint, shoulder and elbow). Each controller consisted of a PID feedback controller combined with an artificial neural network (ANN) arranged as feedforward control (Fig. 3). The ANN provides a way for the controller to learn a non-linear inverse model of the arm. Thus, it is assumed that the learned dynamic covers both, the musculoskeletal responses to the FES and the effects of the shoulder-elbow inter-joint biomechanical coupling. Contrary to past solutions (e.g. ILC [13]), there is no need to take into account this coupling explicitly facilitating the implementation of the controller. This learning process in the ANN occurs by using the output of the PID controller as the correction factor. While the inverse dynamic has not been learned, the PID controller is the main contributor of the control action with a small influence from the ANN. As the movement is repeated and the inverse dynamic is learned, the contributions to the control action are gradually inverted. In the end, the ANN drives the execution of the reaching task while the PID controller compensates only for unknown or unlearned dynamics of the system (e.g. unexpected muscle responses to FES) [16].

A PID controller with an additional inner loop that prevents the integral term to windup was implemented. This additional loop was introduced because only positive output values generate muscle activations (FES assistance) while negative values are ineffective. However, negative values are required for the FEL to learn, which could lead to windup the integral term. Thus, the modified PID controller is given by eq. 1:

1 $$ u(t)= ke(t)+{k}_d\frac{de(t)}{dt}+\int \left({k}_ie(t)+{k}_t{e}_s(t)\right) $$

where *e(t)* represents the error trajectory; *e*_*s*_ *(t)* is the difference between the PID output and the output of the saturator; and *k, k*_*d*_ *, k*_*i*_ and *k*_*t*_ are the constant parameters for the proportional, derivative, integral and the anti-windup terms. To guarantee the correct performance of the PID controller, these parameters were adjusted using the Ziegler and Nichols method of the averaged movement responses in healthy subjects.

The implemented feedforward loop relies on a three-layer ANN (nine input, nine hidden and one output node). A sigmoid function was used to activate neurons in the hidden layer while a linear function was used to activate the output neuron. The inputs to the ANN are the desired angular position, velocity and acceleration profiles, from time *n* to *n + 2,* which result in 9 inputs. These profiles were calculated beforehand (see Eq. 2) and normalized in the range of −1 to 1. The learning process was active along the execution of each movement using the gradient descent algorithm [22]. The ANN size and topology were chosen based on previous studies [16, 18, 23]. In this regard, the ANN size was set as the minimum number of nodes ensuring a proper performance of the system.

The muscular response to FES depends on several factors, such as the placement of the electrodes over the skin and changes in human motor physiology [24]. To avoid bias between inter-session data, the experiments were carried out without previous knowledge of the musculoskeletal system. Thus, the weights of the ANN were initialized to small random values close to zero at the start of all sessions.

#### Reference generator

Studies in the field of motor control showed that arm reaching movements tend to follow a homogeneous pattern across subjects [25]. This pattern is based on a straight path of the hand with smooth and bell-shaped velocity profile. Therefore, to generate such tracking reference, the minimum jerk trajectory method described by Flash and Hogan was implemented [26]. This reference has been successfully used in previous rehabilitation robotic devices [25].

Eq. 2 shows the analytical expression used to derive the position reference required at the input of the FEL control algorithm:

2 $$ {\varnothing}_{r,i}={\varnothing}_i^s+\left({\varnothing}_i^f-{\varnothing}_i^s\right)\left(10{\left(\frac{t}{d}\right)}^3-15{\left(\frac{t}{d}\right)}^4+6{\left(\frac{t}{d}\right)}^5\right) $$

*Ø*_*i*_^*s*^ and *Ø*_*i*_^*f*^ represent the initial and target angles of the *i*-joint respectively, *d* is the movement total duration and *t* is the current time with *0 ≤ t ≤ d*. The velocity and acceleration profiles can be inferred by the first and second time derivatives of eq. 2.

### Participants and evaluation protocol

All participants received oral and written information about the details of the experiment, and signed a consent form to participate and publish the data collected from the experimentation. All experimental protocols followed the Declaration of Helsinki and were approved by the Clinical Ethics Committee of the Centro Superior de Estudios Universitarios La Salle, Universidad Autónoma de Madrid (CSEULS-PI-106/2016).

The system was assessed with two different experiments. Only healthy subjects participated in the first experiment. This experiment was conceived to test the technical viability of the proposed hybrid rehabilitation system and to verify the learning capability (arm dynamic model) of the FEL controller to successfully drive the arm following the desired shoulder-elbow coordinated trajectory with FES (see Experiment 1). The second experiment was designed to test the usability of the proposed hybrid robotic system in a realistic rehabilitation scenario with brain injury patients (see Experiment 2). Therefore, two sessions with a greater number of arm movements than experiment 1 were planned.

#### Experiment 1

For the first experiment, 12 healthy subjects (7 males, 1 left-handed and aged 27.1 ± 2.78 years old) were recruited. Each participant took part in a single evaluation session. Before starting the experiment, the exoskeleton was adjusted to the arm’s dimensions of the subject. The gravity support level was regulated in such a way that the arm was kept about their thigh in the horizontal plane. Surface electrodes (Pals platinum - rectangle 5 × 5 cm) were attached to the anterior deltoids and triceps muscles. Then the maximum pulse amplitude was determined by increasing gradually the current of the stimulator until a motor response was observed with a comfortable stimulation level perceived by the participant. During this procedure, the PW of the stimulation signal was fixed at 450 μs. To define the maximum range of movement and determine the target position, the maximum electrical stimulation intensity to both muscles was simultaneously applied and the resulting movement was recorded. After analyzing the recording data, the target position was defined as the maximum articular angle achieved at each joint (shoulder and elbow). These maximum angles were used in the minimum jerk function (Eq. 2) to generate user-specific reference trajectories.

After this initial procedure, the participants performed twelve reaching movements driven by the FEL controller. During the execution of these movements, participants were asked to let the FES move their arm and to avoid activating any muscle voluntary. For this test, the visual feedback interface was disconnected. So, the participants did not receive any information about the movements. In all trials, a period of three seconds was used to drive the arm from the starting position to the target. Between movements, the participants had a resting period of approximately 10 s to reduce the effects related to muscle fatigue.

#### Experiment 2

For this experimentation stage, patients with brain injury who met the following inclusion criteria were recruited: patients older than 18 years old, with more than 6 months from the brain injury, with hemorrhagic, ischemic stroke or traumatic brain damage, with cognitive capabilities to follow instructions, with response to electrical stimulation in affected upper-limb muscles. Subjects with any implanted metal in the affected upper limb and with a history of epilepsy episodes and/or pregnancy were excluded from the experiment. Three chronic stroke and one traumatic brain injury subjects (age 35 ± 13.09, full details are provided in Table 1) were recruited. None of the patients had prior experience with rehabilitation therapies based on FES or robotic devices.

**Table 1:** Description of patients participating in the study

Patient	Gender	Age (years)	Diagnosis	Affected side	Time since injury (months)	BI	FIM motor subscale	ULMI
P1	Male	52	Ischemic stroke	Left	13	98	85	25
P2	Female	37	Hemorrhagic stroke	Left	15	91	84	25
P3	Female	30	Traumatic brain injury	Left	12	95	90	23.5
P4	Male	21	Ischemic stroke	Left	12	61	66	25

*FIM* functional independence measure, *BI* Barthel index, *ULMI* upper limb part of motricity index

The functional examination of patients was done using three scales: the functional independence measure (FIM) (ranged from 18 to 126) [27], the Barthel index (ranged from 0 to 100) [28], and the upper limb part of Motricity Index (ranged from 0 to 25) [29]. Patients participated in one evaluation and two experimental sessions. The evaluation session was aimed to assess patients’ conditions, verify their response to FES and explain to them the system operation. The experimental sessions were carried out a week later with a separation of 48 h between them. In these sessions, patients had to perform a tracking task with their affected arm following a reference presented on a screen in front of them. After each movement execution, patients were instructed to place their arm back in the initial position and rest for approximately 10 s before starting a new movement. Similarly to the experiment 1, the stimulation was delivered at the anterior deltoids and the triceps and the same initial procedure was followed to define the FES maximum intensity and the range of movement.

On the first day, the session was composed of 5 assisted runs of 8 movements each, plus one additional run of 3 unassisted (without FES) movements. In the second session, participants carried out 8 assisted runs (8 movements) and one unassisted run (3 movements). Thus, a total of 40 and 56 assisted movements were performed on the first and second sessions, respectively. At the start of each session, the feedforward model was reset.

On the pre-session (a week before the experimental sessions) patient P4 presented good response with no discomfort to FES. Nevertheless, on the first experimental session, he reported experiencing discomfort on the arm when FES was applied. This discomfort could be associated to an increase in hypersensitivity during those days. As consequence, the system could not be used with this subject and he was excluded from the experimental sessions.

### Data analysis

#### Experiment 1

The efficacy of the system to assist in the execution of the reaching movement was assessed using the root mean squared error (RMSE) for each controlled joint (Ø_2_ and Ø_5_). The assistance supplied by the controller was quantified relative to the maximum electrical stimulation. This metric was calculated by dividing the norm of the controller output (PW) by the norm of the maximum stimulation that could be supplied (450 us). Complementary, the FEL capability for learning the inverse dynamic of the controlled limb was assessed using the power ratio (PR), (Eq. 3).

3 $$ {PR}_{ff}=\frac{\sum_{k=1}^N{P}_{ff}}{\sum_{k=1}^N{P}_{fb}+\sum_{k=1}^N{P}_{ff}}\times 100 $$

In this equation, the P_ff_ and P_fb_ are the square value of stimulation intensity (output power) of the ANN and the PID controller, respectively. The PR_ff_ represents the proportion of the ANN output relative to the total controller actuation command. This value should be close to 100% when the ANN has learnt the inverse dynamic of the controlled limbs.

The inter-joint coordination between the shoulder and elbow joints (Ø_2_ and Ø_5_) throughout the execution of reaching movements was assessed using the index of the temporal coordination (TC-index) presented in [30]. This single parameter was proposed to evaluate the temporal coordination between adjacent joints involved in the reaching movement. In brief, to suppress tremor-like oscillation in the angular velocity a recurrent exponential smoothing algorithm to the joint velocity was applied: V_i + 1_ = *a*V_i_ + (1-*a*)*v*_i_, where *v*_i_ is the angular velocity, V_i_ is the smoothed value of velocity, and *a* is a smoothness coefficient. The *a* parameter value was set to 0.75 based on previous evidence [30]. Subsequently, a temporal angle (T angle) was calculated as the angle formed between the downward vertical and a line from the origin (placed at the initial position) to successive data points along the velocity-angle plot (ordinate = angular velocity; abscissa = angular displacement). Finally, the TC-index was defined as the difference between the elbow and shoulder T angles at each time throughout the reaching movement. Here, the root mean squared of the TC-index difference between the generated reference and the arm trajectories was calculated to evaluate the capability of the FEL controller to improve the inter-joint coordination.

The mean values of the RMSE, FES intensity, PR_ff_ and the TC-index were calculated across subjects to observe the evolution of these values along the twelve trials executed. Additionally, the RMSE and the PR_ff_ at each joint (shoulder and elbow), and the TC-index score of all users (*n* = 12) on trials one, four, eight and twelve were compared independently using the Friedman’s ANOVA test. Only these trials were selected in order to gain statistical power and considering the symmetry distribution of these trials with respect to the number of repetitions performed. A post hoc analysis of these metrics was conducted applying a Bonferroni correction for significance level (fixed at *p* < 0.0083) and using the Wilcoxon signed-rank tests.

#### Experiment 2

For the experimentation with brain injury subjects, the RMSE at the assisted joints (Ø_2_ and Ø_5_) was averaged for each run and user. The trend of these errors was calculated applying the best-fitting linear regression across the RMSE data of all subjects. A total of 4 linear curves were generated for each combination of subjects, session and joint. Similarly, the PR_ff_ of the FEL controller was averaged for each user and session over the executed run to visualize its evolution along the sessions.

The index of the task performance displayed on the user’s screen during the execution of the task is also analyzed. The following steps were followed to calculate this metric (see Eq. 4). First, the Euclidian distance between the reference trajectory and the actual assisted joint angles during FES application was calculated. Then, the actual Euclidian distance was divided by the maximum distance (reference trajectory vs initial position). This result was subtracted from 1 and multiplied by 100, where a performance of 100 corresponded to perfect tracking.

4 $$ Performance=\left(1-\frac{\sum_{i=1}^T\sqrt{{\left({\varnothing}_{r2,i}-{\varnothing}_{2,i}\right)}^2+{\left({\varnothing}_{r5,i}-{\varnothing}_{5,i}\right)}^2}}{\sum_{i=1}^T\sqrt{{\left({\varnothing}_{r2,i}-{\varnothing}_{2,1}\right)}^2+{\left({\varnothing}_{r5,i}-{\varnothing}_{5,1}\right)}^2}}\right)\times 100 $$

In this equation, *T* is the duration of the movement, Ø_r,i_ is the reference trajectory and Ø_i_ represents the shoulder and elbow joint angles, respectively. The trend of the performance was estimated applying the best-fitting linear regression across the data of all subjects. Two linear curves were generated, each corresponding to one of the two sessions.

In order to analyze the importance of the system’s adaptive assistance to accomplishing accurate reaching movement and to improve the inter-joint coordination, the execution of the unassisted run (3 trials without FES) was compared with the last 3 trials of the final assisted run (with FES). The task’s performance and the TC-index (explained in previous section) were used to compare both conditions. Differences were assessed using the Friedman’s test. Additionally, the post hoc analysis with Wilcoxon signed-rank tests was conducted with Bonferroni correction, resulting in a significance level of *p* < 0.0083.

The satisfaction of the patients after participating in the experimental sessions was assessed using the Quebec User Evaluation of Satisfaction with Assistive Technology 2.0 (QUEST). QUEST is an evaluation specifically designed to measure satisfaction with a broad range of assistive technology devices in a structured and standardized way [31]. The scoring method rated from 1 (not satisfied at all) to 5 (very satisfied). Complementarily, the users’ affective experience with the hybrid system throughout the sessions was evaluated using the Self-Assessment Manikin (SAM). This scale is a non-verbal pictorial assessment technique that directly measures the pleasure, arousal, and dominance associated with a person’s affective reaction to a wide variety of stimuli [32]. All patients were asked to fill both satisfaction surveys after completing the last session.

## Results

### Experiment 1

**Fig. 4:**
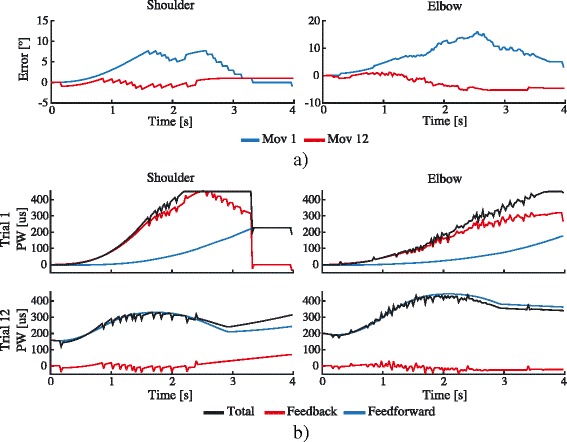
A representative example of the FEL controller performance for user 1. **a** The tracking error during trial 1 (blue) and trial 12 (red) for shoulder (left) and elbow (right) joints. **b** The output signal (pulse width -PW-) of the feedback error learning controller during the first (upper row) and twelfth (lower row) movement execution. Feedback (red) is the control signal given by the feedback controller; Feedforward (blue) represents the control action of the feedforward controller; Total (black) corresponds to the total control signal (PW)

Figure 4 shows a representative example of the FEL operation with one healthy volunteer. The tracking error for shoulder and elbow joints during the first and twelfth trials is depicted in Fig. 4a. In this case, the achieved RMSE in the first trial (blue line) was 4.3° and 8.6° for the shoulder and elbow respectively. While in trial 12, the tracking error was reduced to 0.8° and 3.5° for each joint respectively. Figure 4b depicts the output signal (stimulation PW) of the FEL controller. The first row represents the applied stimulation PW during the first movement attempt. In this case, the total assistance (black line) is mostly overlapped with the contribution of the feedback controller (in red), resulting in a PR_ff_ (contribution of ANN in blue) of 19% and 10% for shoulder and elbow assistances, respectively. The contribution of each controller is swapped on trial 12 as depicted in the second row of the same figure. At this point, the feedforward contribution increased, with a PR_ff_ of 98 and 99% for each joint, while the feedback controller was only compensating for disturbances.

**Fig. 5:**
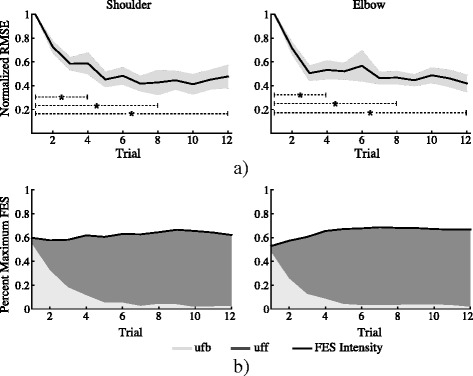
**a** Mean values of the normalized root mean squared error (RMSE, black line) and its standard error (gray shaded areas) across healthy subjects, corresponding to the shoulder (left) and elbow (right) joints. Dotted lines denote significance difference between trials. **b** Mean values of provided FES intensity, represented as a percent of the maximum stimulation intensity, across subjects. Light gray and dark gray areas depict the contribution of the feedforward (ufb) and feedback (uff) loop to the total FES intensity, measured with the power ratio (PR)

Figure 5a shows the mean of the normalized RMSE score with respect to the first trial across subjects over the 12 reaching trials and their correspondent standard error (shaded areas). A final score of 0.47 and 0.41 for each joint respectively was achieved at the last trial (12th movement), indicating an error reduction of more than 50% with respect to the first trial execution. When analyzing tracking accuracy for the first, fourth, eighth and twelfth trials (values shown in Table 2), the Friedman’s ANOVA test revealed that the RMSE along these trials differed significantly in both joints, with χ^2^(3) = 14.7, *p* = 0.002 and χ^2^(3) = 21.5, *p* < 0.001 for shoulder and elbow joints respectively. The post hoc analysis (results on Table 3) uncovered that for both joints, the RMSE value for trial four, eight and twelve were significantly reduced when compared with the trial one. The differences between trials four, eight and twelve were not significant in any joints.

**Table 2:** Mean and standard deviation values across healthy subjects

	RMSE [°]		Power Ratio [%]		Normalized RMS TC-index
	Shoulder (Ø_2_)	Elbow (Ø_5_)	Shoulder (Ø_2_)	Elbow (Ø_5_)	
Trial 1	5.9 ± 2.3	12.1 ± 3.9	6.9 ± 7.9	8.22 ± 4.7	1
Trial 4	3.5 ± 2.7	6.5 ± 4	80.7 ± 17.1	86.3 ± 14.2	0.93 ± 0.31
Trial 8	2.9 ± 3.7	5.3 ± 1.7	92.8 ± 13.9	94.5 ± 10.4	0.83 ± 0.46
Trial 12	3.2 ± 3.6	4.9 ± 3.1	95.6 ± 7.3	96.9 ± 5.3	0.70 ± 0.38

*RMSE* root mean squared error, *RMS* root mean square, *TC-index* temporal coordination index

**Table 3:** Results of the Wilcoxon post hoc test

	RMSE			PR				
	1st vs 4th trial	1st vs 8th trial	1st vs 12th trial	1st vs 4th trial	1st vs 8th trial	1st vs 12th trial	4th vs 8th trial	4th vs 12th trial
Shoulder (Ø_2_)	*p* = 0.002 *r* = −0.62	*p* = 0.005 *r* = −0.57	*p* = 0.003 r = −0.6	*p* < 0.001 *r* = −0.71	*p* < 0.001 *r* = −0.71	*p* < 0.001 *r* = −0.71	*p* > 0.008	*p* = 0.002 *r* = −0.62
Elbow (Ø_5_)	*p* = 0.001 *r* = −0.67	*p* < 0.001 *r* = −0.71	*p* < 0.001 *r* = −0.71	*p* < 0.001 *r* = −0.71	*p* < 0.001 *r* = −0.71	*p* < 0.001 *r* = −0.71	*p* = 0.005 *r* = −0.57	*p* < 0.001 *r* = −0.71

Bonferroni correction for multiple comparison established the level of significant at *p* < 0.0083. p: level of significance; r: effect size; *RMSE* root mean squared error, *PR* power ratio

The FES intensity, expressed as a percentage of the maximum stimulation, applied at shoulder and elbow joints over the twelve trials execution is shown in the Fig. 5b. Here, the total assistance is given by the contribution of the feedforward (dark gray area) and feedback (light gray area) controllers, which are measured using the PR score. In both joints, it can be observed that the PR_ff_ is increased as the movement is repeated (dark gray area), while the output of the feedback loop (PR_fb_) is decreased (light gray area). The statistical test found that the PR_ff_ at trials one, four, eight and twelve (values shown in Table 2) differed significantly in both joints, with χ^2^(3) = 29.5, *p* < 0.01 for shoulder and χ^2^(3) = 32.7, *p* < 0.001 for the elbow. The post hoc multiple comparison showed that in both joints, the contribution of the feedforward controller (PR_ff_) at trials four, eight and twelve increased significantly when compared with the value at the first trial and the twelfth trial with respect to the fourth (results of post hoc analysis are shown in Table 3). At the elbow joint, the PR_ff_ value for trial eight was also significantly higher than the fourth trial, but not at the shoulder joint. No significant differences were observed between trials eight and twelve in any joints.

**Fig. 6:**
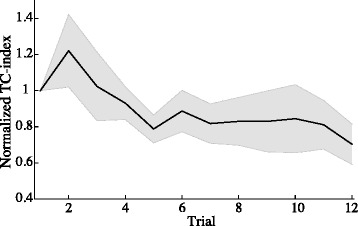
Evolution of the root mean square error of the temporal coordination (TC) index between the generated reference and arm trajectories. Shaded area represents standard error

The normalize RMS evolution of the TC-index between the reference and the arm trajectories considering the shoulder and elbow joints during the execution of reaching movements is presented in Fig. 6. It can be also observed that the inter-joint coordination index is reduced across the executed movements. Although the statistical test did not find significant differences between trials one, four, eight and twelve (χ^2^(3) = 6.7, *p* = 0.08), the final score of the TC-index (0.7 ± 0.4) shows an improvement 30% with respect to the first trial (see last column of Table 2).

### Experiment 2

#### Performance results

**Fig. 7:**
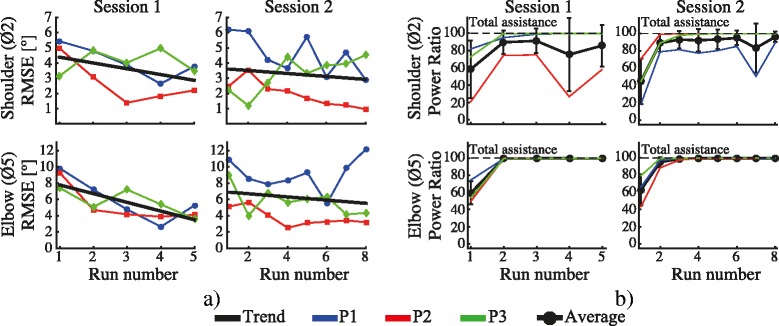
**a** Evolution of the root mean square error (RMSE) averaged for each run. The first column represents the RMSE for session one divided in shoulder (top) and elbow (bottom), while the second column depicts the error for session two. The black lines represent the calculated linear regression for each combination of subject, muscle and session. **b** Evolution of the PR_ff_ for each subject. The black line represents averaged PR_ff_ across subjects and its corresponding standard error. The first column represents the PR_ff_ for session one divided in shoulder (top) and elbow (bottom), while the second column depicts the value corresponding for session 2

Figure 7a illustrates the evolution of the RMSE as function of the executed run for each subject, joint and session. The estimated linear fitting curves at the shoulder resulted in slopes of −0.38 for session one, and −0.1 for session two. These results represent an average RMSE reduction from 4° to 2.9°. While the fitting for the elbow presented a slope of −1.07 and −0.2 for each session respectively, which corresponds to a decrease in the RMSE value from 7.3° to 4.5°. Figure 7b shows the evolution of the PR_ff_ over the runs for each participant, muscle and session (blue, red and green curves). Additionally, the corresponding average values across subjects and its corresponding standard deviation are represented (black lines). At the shoulder joint, the PR_ff_ presented an average value across subjects of 58.3 ± 33.1% and 44.8 ± 26.2% on the first run for session one and two respectively. This value has increased to 89.5 ± 13.4% and 89.1 ± 10.1% on the second run for each session respectively. No important differences were observed on the remaining runs. For the elbow joint, the PR_ff_ value at the elbow presented an increasing trend with an average value across subjects of 59.4 ± 13.2% and 62.9 ± 18.3% on the first run for session one and two respectively. This value was increased to 99.4 ± 0.3% and 95.2 ± 6.9% on the second run for each session, and it achieved a final value of 99.6 ± 0.2% and 99.8 ± 0.1% on the last executed run of each session respectively.

**Fig. 8:**
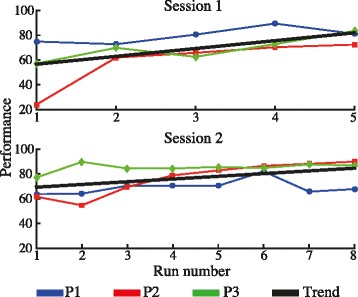
Averaged tracking performance for each run corresponding to session one and two. The black lines represent the linear fitting regression across subjects

Figure 8 depicts the corresponding averaged tracking performance for each run during session one and two. The black lines represent the trend of these values for each session. In both cases (session 1 and 2), the linear curves present positive slopes (0.06 for session one and 0.02 for session two) indicating an increase in performance from 56% to 82%, and from 69% to 84% for each session, respectively.

**Fig. 9:**
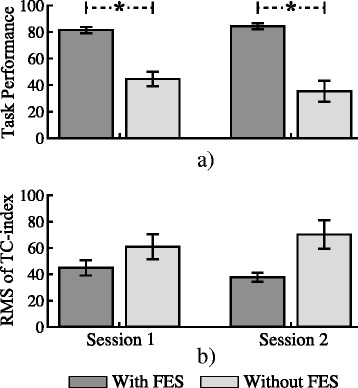
**a** Mean task’s performance across subjects considering the last assisted and the unassisted runs. The error bars represent the standard error; **b** Mean root mean squared (RMS) of the temporal coordination index (TC-index) differences between the generated reference and the arm trajectories across subjects considering the last assisted and the unassisted runs. The error bars represent the standard error. Asterisks indicate significant differences

Figure 9a shows the quantitative comparison of the task’s performance when the movement was carried out with and without FES. This picture illustrates the average values of the task’s performance across all users, where the error bars represent the standard error. The Friedman’s ANOVA reveals a significant difference between conditions (χ^2^(3) = 22.2, *p* < 0.001). The post host confirm that in both session, the task’s performance was significantly better when participants carried out the reaching task with FES assistance (session one: 81.4 ± 9% and session two: 84.2 ± 9%) than when participant performed the task without FES (sessions one 44.4 ± 18.9% and two 35.3 ± 25.8%), with *p* = 0.008, *r* = −0.63 for the first and *p* = 0.004, *r* = −0.68 for the second session respectively. No significant difference was found in the score between neither the unassisted task nor the assisted task on different sessions, meaning that both conditions (assisted and unassisted) did not change between sessions. The RMS values of the TC-index between reference and the arm trajectories (shoulder and elbow) for the assisted and the unassisted runs across subjects are depicted in Fig. 9b. Although the execution of reaching movement without FES presented worst inter-joint coordination (higher RMS of the TC-index difference between the reference and the arm trajectory) in both sessions, the statistical test did not find significant differences between these values and the achieved with FES.

#### Satisfaction assessment

Table 4 shows the results of the satisfactions scales. In the QUEST questionnaire, the system obtained a high evaluation score in all the items. This assessment reveals an overall average score of 34.67 over the 35 points. The SAM survey scored an overall average value of 9 in pleasure, 8.33 in dominance, while the arousal item was set slightly over the middle of the scale with a value of 5.33.

**Table 4:** Satisfaction score for all patients

	P1	P2	P3	Mean
* Quest. How satisfied are you with the system features?*				
1. The dimensions (size, height, length, width) of your assistive device?	5	5	5	5
2. The weight of your assistive device?	5	5	5	5
3. The easy in adjusting (fixing, fastening) the parts of your assistive device?	5	5	5	5
4. How safe and secure your assistive device is?	5	5	5	5
5. How easy it is to use your assistive device?	5	5	5	5
6. How comfortable your assistive device is?	4	5	5	4.67
7. How effective your assistive device is (the degree to which your device meets your needs)?	5	5	5	5
* SAM assessment.*				
1. Pleasure	9	9	9	9
2. Arousal	5	6	5	5.33
3. Dominance	9	9	7	8.33

*QUEST* Quebec User Evaluation of Satisfaction with Assistive Technology 2.0, *SAM* Self-Assessment Manikin. QUEST scale: 5 (very satisfied), 4 (satisfied), 3 (more or less satisfied), 2 (not very satisfied) and 1 (not satisfied at all). SAM depicts the pleasure, arousal and dominance dimension with a graphic character arrayed along a continuous nine-point scale

## Discussion

This study evaluated the usability of a hybrid robotic system for the rehabilitation of reaching movements in patients with upper limb motor impairment due to a brain injury. The proposed hybrid platform integrates several subsystems bringing about a comprehensive self-contained tool. The system provided an adaptive assistance through the implementation of the FEL controller. This is the first time that the FEL algorithm was integrated in a hybrid robotic system to assist in two simultaneous muscles during the execution of a functional task such as reaching. It is also the first time the FEL has been tested with brain injury patients using a hybrid robotic system for upper limb rehabilitation.

### Technical viability and system performance

Experiment 1 was aimed at showing that proposed hybrid robotic system is able to drive the execution of reaching movements by activating the shoulder and elbow joints simultaneously using only FES. Healthy subjects were asked to refrain from activating their muscles voluntary and let the FES move their arm. In this experiment, the capacity of the FEL controller to learn from the tracking errors in order to adjust the control action according to the individual responses to FES was demonstrated. The significant reduction of the RMSE at both joints (Fig. 5a, lines in black) confirms an improvement of the tracking accuracy as the movement is repeated. The shaded areas in dark gray depicted in Fig. 5b denote the significant increase of the PR_ff_ measures in the controlled joints (Ø_5_ and Ø_2_), showing the learning process of the controller. Furthermore, as no significance was found for RMSE after the fourth trial and in the PR_ff_ after the eight trial with the healthy participants, it can be suggested that the FEL only requires a few movement examples to attain a stable and appropriate assistance.

Similar behavior was demonstrated in Experiment 2 with brain injury participants. In this experiment, brain injury patients were asked to realize the movement actively while the FES provided the activation needed for the patient to complete the task. The negative slopes of the linear fitting curves derived from the RMSE values over the two sessions (Fig. 7a) show a trend towards a reduction of the error as the tracking task was repeated. This improvement was translated in an enhancement of the performance score fed back to the users (Fig. 8). In general, an improvement in the performance of the tasks from 62.5% to 83% (averaged from both sessions) was achieved. The PR_ff_ values after the second executed run also presented important higher score than the first run in both joints. The score obtained during the execution of the task with and without FES assistance revealed the difficulty of patients to carry out the required reaching movement without FES (see Fig. 9). The average task’s performance of the users during the execution of the assisted reaching task (82.8%, averaged from both sessions) was twice of the value obtained without assistance. Therefore, the results confirmed that the hybrid assistance improved significantly the task’s performance by adapting the delivered FES intensity according to the patients’ needs and capabilities, helping them to complete the tracking movements.

When looking at the effect of the FEL controller to the inter-joint coordination during the execution of reaching movement, from experiment 1 can be observed an improvement of the shoulder-elbow coordination throughout the trials execution (see Fig. 6). However, this improvement did not result into a significant improvement when compared with the first trial. Similarly, experiment 2 showed that assisted movement resulted in better inter-joint coordination than the movement performed without assistance (without FES). These results suggest the capability of the FEL controller to learn the shoulder-elbow inter-joint biomechanical coupling.

Freeman et al. in [14] presented the use of ILC to continuously adapt the FES intensity during reaching movements in a similar hybrid robotic system. This algorithm was tested with healthy subjects performing ten repetitions of a given task. The RMSE reported in their study was, on average for the first six trials, 9.69° ± 9.22° and 12.54° ± 9.87 for shoulder and elbow angles, respectively. In comparison, the approach presented here achieves an overall tracking error of 3.2° ± 3.6° for the shoulder and 4.9° ± 3.1° for the elbow after 12 trials in the experiment with healthy subjects (*n* = 12). These improvements could be attributed to the FEL capability of learning a more precise inverse dynamics model of the non-linear musculoskeletal characteristics of the arm [16, 33]. Therefore, the proposed FEL system represents a robust and reliable strategy to tackle the subject’s individual differences and the necessity of a complex model describing the arm dynamic for 3D movement [13, 14] (e.g. identifying the model’s parameters, and errors in the model due to unmodelled dynamics and model linearization).

Model-based controllers typically require the definition of multiple parameters before their use, resulting in time-consuming tasks and requiring one or more additional sessions prior to the intervention [7, 13, 34]. Moreover, due to the physiological changes occurring over the days, a re-calibration procedure is often required to maintain the performance of these approaches [34]. Unlike model-based systems, the FEL strategy do not need a user-specific model nor a previous model, the algorithm is always learning and adapting in real time. With the approach proposed here, there is no need to adjust any parameters within one session, between sessions or between patients, which provides great robustness to the rehabilitation, especially if it is to be used by clinical (non-technical) operators.

### User satisfaction

The users’ perception when dealing with FES or robotic technologies for upper extremity rehabilitation is scarcely reported in the literature. Nevertheless, if a system is not found useful and motivational, it will be used less frequently and adherence will be an issue [35]. In this regard, QUEST user’s satisfaction scale reported great satisfaction with the system in all items, since most scores reached a value of 5 over 5. The SAM scale results related to pleasure and arousal showed scores of 9 and 5,33 ± 0,58, respectively, suggesting that patients were satisfied with the use of the system. The result of dominance shows that patients perceived high level of control (9/10) while using the system. A low score in dominance may be interpreted as a marker of patients’ feeling of being controlled or submissive, adopting a passive attitude.

Patients’ motivation has been shown to be an important predictor of long-term changes in quality of life and rehabilitation outcomes [36]. The QUEST and SAM assessments suggest that patients found the system attractive, and they adopted an active attitude without feeling under pressure or stressed.

### Limitations of the study

Due to the complexity of the shoulder movement during the execution of reaching movements in unconstrained space [37], bigger variability in the PR_ff_ metric are observed at this joint when compared with the elbow. This effect is more noticeable with brain injury patients (see Fig. 7b). This larger variation can be attributed to a more varying response at the shoulder joint to FES. As no mechanical assistance is provided during the movement execution, these differences can be explained by the amount of electrical current required at the shoulder to lift the arm up. Meadmore et al. observed similar limitations in [13]. Therefore, the use of mechanical devices with active actuator could result in a more consistent response at the shoulder joint. Still, the development of an optimally shared control between the FES and the mechanical assistance are needed [4].

The use of the proposed system could be limited by different condition of a patient. In the present study, the participant P4 had to be excluded from the experiment due to a change in his perception of FES, possibly due to a hypersensibility experienced throughout the experimental sessions. Therefore, special attention should be paid to requirements for participant selections refining the inclusion criteria. Certainly, the guidance given by Huang et al. in [38] can be followed, where it was suggested that patients with medium level (suggested by Fugl-Meyer Assessment and Motor Assessment Scale score) of motor skills are preferred when considering robot-based rehabilitation therapies. Alternatively, active exoskeleton could be considered to reduce the needed FES stimulation intensity. With such a system, the exoskeleton assistance can be reduced progressively to increase the FES stimulation and promote voluntary movement.

The results reported in this study are based on a reduced number of patients and sessions. As the potential rehabilitation benefits of the present hybrid robotic system is out of the scope of this study, it is necessary to conduct a larger clinical study involving more patients and sessions.

## Conclusion

A hybrid robotic system for rehabilitation of reaching movement was presented. The system is comprised of several subsystems that cooperatively carry out the rehabilitation exercise. A feedback error learning controller was integrated into the platform to learn the inverse dynamic model of the arm and adjust the level of assistance according to the user capabilities. The usability of the hybrid system has been proved through the experiments carried out with healthy participants and patients with a brain injury. The study demonstrated the capability of FEL scheme to assist the execution of reaching movements in 3D space. Patients’ reports on the received intervention revealed a great satisfaction and acceptance of the hybrid robotic system. These results support the idea that complementing rehabilitation with the hybrid system proposed here may be useful to increase the dosage of therapy and to augment patient’s engagement and motivation during the rehabilitation process.

## Abbreviations

ANN: Artificial neural network
DoF: Degree of freedom
FEL: Feedback error learning
FES: Functional electrical stimulation
HLC: High-level controller
ILC: Iterative learning control
PID: Proportional-integral-derivative
PR: Power ratio
PW: Pulse width
QUEST: Quebec user evaluation of satisfaction with assistive technology
RMS: Root mean square
RMSE: Root mean square error
SAM: Self-assessment Manikin
TC-index: Temporal coordination index
